# Smac mimetics LCL161 and GDC-0152 inhibit osteosarcoma growth and metastasis in mice

**DOI:** 10.1186/s12885-019-6103-5

**Published:** 2019-09-14

**Authors:** Tanmay M. Shekhar, Ingrid J. G. Burvenich, Michael A. Harris, Angela Rigopoulos, Damien Zanker, Alex Spurling, Belinda S. Parker, Carl R. Walkley, Andrew M. Scott, Christine J. Hawkins

**Affiliations:** 1 Department of Biochemistry and Genetics, La Trobe Institute for Molecular Science, La Trobe University, Bundoora, Victoria 3086 Australia; 2 Tumour Targeting Laboratory, Ludwig Institute for Cancer Research and Olivia Newton-John Cancer Research Institute, Melbourne, Australia; 3 School of Cancer Medicine, La Trobe University, Melbourne, Australia; 4 St. Vincent’s Institute, Fitzroy, Victoria 3065 Australia; 5 Department of Medicine, St. Vincent’s Hospital, University of Melbourne, Fitzroy, Victoria 3065 Australia; 6 Mary MacKillop Institute for Health Research, Australian Catholic University, Melbourne, Victoria 3000 Australia; 7 Departments of Medical Oncology and Molecular Imaging & Therapy, Austin Health, Heidelberg, Melbourne, Australia; 8 Department of Medicine, University of Melbourne, Melbourne, Australia

**Keywords:** Osteosarcoma, Bone cancer, Sarcoma, Smac mimetic, IAP antagonist, Metastasis, Anthracycline, Mouse cancer model, Targeted therapy

## Abstract

**Background:**

Current therapies fail to cure over a third of osteosarcoma patients and around three quarters of those with metastatic disease. “Smac mimetics” (also known as “IAP antagonists”) are a new class of anti-cancer agents. Previous work revealed that cells from murine osteosarcomas were efficiently sensitized by physiologically achievable concentrations of some Smac mimetics (including GDC-0152 and LCL161) to killing by the inflammatory cytokine TNFα in vitro, but survived exposure to Smac mimetics as sole agents.

**Methods:**

Nude mice were subcutaneously or intramuscularly implanted with luciferase-expressing murine 1029H or human KRIB osteosarcoma cells. The impacts of treatment with GDC-0152, LCL161 and/or doxorubicin were assessed by caliper measurements, bioluminescence, ^18^FDG-PET and MRI imaging, and by weighing resected tumors at the experimental endpoint. Metastatic burden was examined by quantitative PCR, through amplification of a region of the luciferase gene from lung DNA. ATP levels in treated and untreated osteosarcoma cells were compared to assess in vitro sensitivity. Immunophenotyping of cells within treated and untreated tumors was performed by flow cytometry, and TNFα levels in blood and tumors were measured using cytokine bead arrays.

**Results:**

Treatment with GDC-0152 or LCL161 suppressed the growth of subcutaneously or intramuscularly implanted osteosarcomas. In both models, co-treatment with doxorubicin and Smac mimetics impeded average osteosarcoma growth to a greater extent than either drug alone, although these differences were not statistically significant. Co-treatments were also more toxic. Co-treatment with LCL161 and doxorubicin was particularly effective in the KRIB intramuscular model, impeding primary tumor growth and delaying or preventing metastasis. Although the Smac mimetics were effective in vivo, in vitro they only efficiently killed osteosarcoma cells when TNFα was supplied. Implanted tumors contained high levels of TNFα, produced by infiltrating immune cells. Spontaneous osteosarcomas that arose in genetically-engineered immunocompetent mice also contained abundant TNFα.

**Conclusions:**

These data imply that Smac mimetics can cooperate with TNFα secreted by tumor-associated immune cells to kill osteosarcoma cells in vivo. Smac mimetics may therefore benefit osteosarcoma patients whose tumors contain Smac mimetic-responsive cancer cells and TNFα-producing infiltrating cells.

## Background

Osteosarcoma is the most common primary bone malignancy. These genomically unstable cancers develop due to oncogenic transformation, usually involving inactivation of p53 [1], of osteoblast lineage cells or their mesenchymal progenitors [2, 3]. Osteosarcomas typically arise in the extremities of teenagers. Osteosarcoma is rarer in older populations, and approximately half of elderly osteosarcoma patients acquire these cancers secondarily to Paget’s disease or bone irradiation [4]. Osteosarcoma preferentially metastasises to the lungs, and around a fifth of patients have detectable metastases at diagnosis [5, 6].

Interventions for osteosarcoma patients typically involve chemotherapy (usually methotrexate, doxorubicin and cisplatin) before and after amputation or limb-sparing surgery [7]. The introduction of chemotherapeutics to osteosarcoma treatment regimens in the 1970s and 1980s improved 5-year osteosarcoma survival rate from ~ 20% in the 1960s to ~ 60% by the 1980s [8], however there has been no significant improvement since [9], and current treatments are only effective for 20–30% of patients with metastatic disease [6, 9]. Better therapies are needed for non-responsive tumors. Various targeted therapeutic agents such as inhibitors of VEGFR, IGF1-R, mTOR, and immune checkpoint molecules, are presently being evaluated clinically for osteosarcoma [10].

“Smac mimetics” (also known as “IAP antagonists”) are small molecules developed to mimic the activity of the cellular protein Smac [11, 12]. They induce cell death by inhibiting the activity of pro-survival IAP proteins such as XIAP, cIAP1 and cIAP2 [13]. XIAP exerts its pro-survival activity through inhibition of pro-apoptotic caspase-3, − 7 and − 9 [14], and some IAP antagonists can relieve this inhibition by binding to XIAP. On the other hand, cIAP1/2 polyubiquitinate RIPK1, ultimately promoting NF-κB-mediated induction of genes that induce cell proliferation, migration and invasion in cells exposed to TNFα [15]. Smac and its mimetics promote cIAP1/2 auto-ubiquitination and degradation, which leads to de-ubiquitination of RIPK1, resulting in formation of the “ripoptosome” complex [16]. The pro-apoptotic protein caspase-8 is activated in this complex to induce cell death through activation of executioner caspases, if their inhibition by XIAP is relieved [16]. RIPK1 can also activate RIPK3 and MLKL to induce necroptosis, a form of caspase-independent cell death [17] that can be activated by TNFα in cells lacking caspase-8 and IAP activity [18].

Monovalent Smac mimetics, such as GDC-0152 [19] and LCL161 [20, 21], resemble the amino terminus of Smac, and can interact at one site of an IAP protein, whereas bivalent compounds like Birinapant [22] target two such sites conferring higher potency and affinity. Smac mimetics also differ in their affinities towards particular IAP proteins. Birinapant preferentially binds to cIAP1 and cIAP2 [22], however LCL161 and GDC-0152 bind with similar affinities to XIAP, cIAP1 and cIAP2 [19, 20]. Smac mimetics can induce cell death in some cell types as sole agents, through stimulation of the non-canonical NF-κB pathway to produce TNFα, which then stimulates TNFR1-mediated cell death pathways [23–25]. Other cells types, including osteosarcoma cells [26], fail to produce autocrine TNFα and therefore are only efficiently killed by Smac mimetics when exposed to exogenous TNFα.

Smac mimetics have been shown to be well-tolerated in patients, however high doses of LCL161 triggered cytokine release syndrome due to autocrine TNFα production [20], and occasional patients administered Birinapant experienced Bell’s Palsy [27, 28]. As single agents, Smac mimetics induced complete or partial remissions in a minority of patients and stabilized disease in others [29]. Over a third of acute myeloid leukemia patients administered DEBIO1143 with chemotherapy experienced complete remissions, although half subsequently relapsed [30]. Pre-clinical studies revealed that Smac mimetics could also augment the cytotoxicity of other targeted therapies [22, 31–43]. The utilities of some of these co-treatments are presently being assessed in clinical trials. As mentioned above, exposure to Smac mimetics only provokes autocrine TNFα production to facilitate sole agent killing in cells from a subset of tumors. This does not necessarily preclude effective Smac mimetic-based treatment of tumors composed of such cells though, as Smac mimetics can boost systemic TNFα levels, conceivably providing sufficient TNFα at the tumor site to enable Smac mimetics to activate cell death pathways. Oncolytic viruses that stimulated intratumoral inflammatory cytokine production synergized strongly with Smac mimetics in mouse models of glioblastoma, rhabdomyosarcoma, mammary carcinoma and colon cancer [44–47]. Cooperation by inflammatory cytokines and Smac mimetics has been documented to stimulate anti-tumor immunity via both innate and adaptive mechanisms [48, 49]. Indeed, Smac mimetics enhanced the efficacy of immune checkpoint inhibitors in mice [47], even in a context in which the tumor cells lacked cIAP1 and 2 [50].

There have been very limited investigations into the possible utility of Smac mimetics for treating osteosarcoma, with no clinical trials registered or conducted to date, however several lines of evidence suggest that these agents may be efficacious for this malignancy. The major molecular targets of these drugs, cIAP1 and 2, have been documented to be upregulated in osteosarcoma, and their silencing impaired osteosarcoma growth in mice [51]. A subset of Smac mimetics (SM-164, LCL161 and GDC-0152) potently cooperated with TNFα to kill cells from many murine osteosarcomas in vitro, and this toxicity was potentiated by co-treatment with doxorubicin [26]. Other studies have also reported the sensitivity of osteosarcoma cells to SM-164 [52], GDC-0152 [53] and DEBIO1143/AT-406 [54] in vitro. So far, only two articles have reported effects of Smac mimetics on osteosarcomas in vivo. DEBIO1143, a Smac mimetic that exhibited poor anti-osteosarcoma in vitro [26], did not significantly affect the growth of KHOS/NP cells implanted into nude mice as a sole agent [54]. Co-treatment with doxorubicin yielded a slight but statistically significant reduction in tumor growth a week after treatment began, although that effect’s duration was not reported [54]. The other in vivo study examined the anti-osteosarcoma efficacy of LCL161, which was one of the most active Smac mimetics in vitro [26]. Disappointingly, those authors observed that LCL161 treatment only slightly reduced the growth of human osteosarcoma xenografts in SCID mice [21]. However, SCID mice have lower levels of TNFα than wild type mice [55], and since osteosarcoma cells were only sensitive to Smac mimetics in vitro when co-treated with TNFα [26], the SCID xenograft model may underestimate the efficacy of LCL161. Levels of TNFα within osteosarcomas have not been previously reported, but published data suggest that they may be high. Serum TNFα levels were documented to be elevated in osteosarcoma patients, with concentrations reflecting disease progression and primary tumor size [56, 57]. Osteosarcomas harbor a large population of macrophages [58–60] that could secrete TNFα, and implantation of transformed mesenchymal cells into mice produced osteosarcomas that were infiltrated by TNFα-expressing macrophages [61]. The observation that osteosarcoma cells were sensitive in vitro to TNFα combined with physiologically achievable concentrations of Smac mimetics, coupled with these suggestions that osteosarcomas may contain high levels of TNFα, prompted us to examine the anti-osteosarcoma activity of selected Smac mimetics in vivo, as sole agents or in combination with doxorubicin, using nude mice implanted subcutaneously or intramuscularly with murine or human osteosarcoma cells.

## Methods

### Animal and cells

Murine 1029H osteosarcoma cells [26] and human osteosarcoma cell lines OS9, OS17 [62] (generated from in vivo-passaged tumors provided by Peter Houghton), SaOS2, U2OS and SJSA1 (provided by Damian Myers) were cultured in αMEM (Lonza, Australia) supplemented with 100 units/ml Penicillin/Streptomycin (Sigma-Aldrich, USA), 2.92 mg/ml L-glutamine (Sigma-Aldrich) and 10% fetal bovine serum (FBS) (Scientifix, Australia). Human OS cells KHOS, KRIB and 143B (provided by Nicholas Saunders) were cultured in DMEM media (Invitrogen, USA) supplemented by 10% FBS. 1029H, KRIB and 143B cells were engineered to express luciferase and mCherry genes through retroviral transduction with a pMSCV-Luciferase-IRES-mCherry plasmid [63]. Phoenix-Eco (ATCC) and PT67 (ATCC) packaging cells were cultured in DMEM media supplemented with 10% FBS. For ex vivo treatments, cells were isolated from tumors as previously described [64] and cultured in the media specified above for 1029H cells. All cells were cultured at 37 °C in air supplemented with 5% CO_2_.

Five to 6 week old BALB/c-Foxn1^nu^/Arc (“nude”) mice were purchased from ARC (Australia). These animals, and *Osx*-Cre p53^*fl/fl*^ pRb^*fl/fl*^ mice [65] and p53^*fl/fl*^ pRb^*fl/fl*^ mice [65] were housed at La Trobe Animal Research Facility in individual ventilated cages, with 12-h light/dark cycling, and unrestricted access to food and water. Mice were monitored and weighed each day. Euthanasia was performed by CO_2_ asphyxiation or cervical dislocation, with or without prior cardiac puncture.

### Tumor implantation and in vivo imaging

For sub-cutaneous implantation, 500,000 luciferase-expressing 1029H cells (1029H-Luc) were resuspended in 200 μl of media and Cultrex Reduced Growth Factor Basement Membrane Matrix (Cultrex) (Trevigen; USA) mixture (1:1) and injected sub-cutaneously into the hind flank of a mouse using a 26-gauge needle. Luciferase-expressing KRIB-Luc cells were implanted intramuscularly in the anterior tibial muscle of mice: under isoflurane-induced anesthesia, 20 μl of a cell suspension containing 50,000 cells in phosphate-buffered saline (PBS) and cultrex (1:1) was injected into the anterior tibial (cranial tibialis) muscle using a 29-gauge insulin syringe. Mice were subjected to bioluminescence imaging using an IVIS Lumina XR III (Perkin Elmer; USA) to monitor tumor growth. Each mouse was injected intraperitoneally with 150 mg/kg of D-Luciferin, Potassium salt (Pure Science, New Zealand), anesthetized using isoflurane and placed on the imaging platform of the IVIS machine. Eight mins after injection, bioluminescence was acquired in 12 segments with 1 min intervals between each segment. A circular region of interest was constructed encompassing the tumor, and luminesce intensity was determined for this region by measuring photons/sec. The highest luminescence measurement recorded within those segments was used as a measure of tumor size for that time point.

### PET/MRI

In vivo PET imaging was performed on three GDC-0152-treated and three control (vehicle-treated) 1029H-Luc tumor-bearing nude mice 9 days after final therapy administration. Mice were fasted for three hours before receiving a dose of 14.8 MBq ^18^F-FDG (Austin Health, Heidelberg, Australia). After injection, mice were anesthetized immediately by inhalation of isofluorane for the duration of the imaging study. Mice were imaged with a nanoScan PET/MR camera (Mediso, Budapest, Hungary). For each animal, Magnetic Resonance Imaging (MRI) acquisition was performed first using a T1-FSE sequence. Positron Emission Tomography (PET) acquisition was performed 1 h after injection, for 15 min. For visualization of ^18^F-FDG uptake in different organs, PET images were decay-corrected using the half-life of ^18^F (109.77 mins) and normalized using the standardized uptake (SUV) factor defined as injected dose (kBq) per g body weight. To calculate ^18^F-FDG SUV uptake in the tumor, regions of interest were drawn in each section to define the volume of interest (VOI, mL) of the tumor in each section. SUV is defined as:

$$ SUV=\frac{C_t\left( kBq/ mL\right)}{\frac{Injected\ Dose\ (kBq)}{Body\ Weight\ (g)}} $$

where C_t_ is the radioactivity concentration in a specific VOI at time t after injection.

### *In vivo* treatments

Mice were ordered on the basis of their tumour bioluminescence, then alternately distributed into the treatment groups to ensure that each group contained mice with a similar range of tumor sizes prior to treatment. Doxorubicin (Sigma-Aldrich) was dissolved and diluted in PBS to achieve concentrations of 0.4 to 0.6 mg/ml. Doxorubicin was injected at 2–6 mg/kg once a week for 4 weeks through tail intravenous injections using 30-gauge needles. GDC-0152 (Genentech, USA) was prepared by dissolving the drug in DMSO at 80 mg/ml, and then diluting to desired concentration using PBS (pH 6.0). LCL161 (Novartis, USA) formulations and working solutions were prepared as previously described [21]. GDC-0152 and LCL161 were administered through oral gavage.

### Cell viability assay

In vitro responses of cells to doxorubicin, GDC-0152, LCL161 and/or murine or human TNFα (Peprotech, USA) were determined by measuring the amount of ATP activity in cells using CellTiter-Glo 2.0 (Promega; USA), as previously described [26].

### Cell and tumor lysis, electrophoresis and immunoblotting

Cells and tumor samples were lysed using RIPA lysis buffer (150 mM sodium chloride, 1.0% Triton X-100, 0.5% sodium deoxycholate, 0.1% SDS, 50 mM Tris, pH 8.0) supplemented with protease inhibitor cocktail (Roche; Switzerland). Tumor samples were homogenized in RIPA lysis buffer using an electrical tissue homogenizer. The lysates were cleared by centrifuging for 15 min at 16,100 g at 4 °C. Total protein was determined using the bicinchoninic acid (BCA) method (Micro BCA Protein assay kit, Thermo Fisher Scientific; USA). Immunoblotting was performed as previously described [26]. Antibodies used in this study were anti-cIAP (MBL Life Science, Japan), mouse anti-actin (Sigma-Aldrich), donkey anti-rabbit-HRP (GE Healthcare Life Sciences; USA) and rabbit anti-mouse-HRP (Sigma-Aldrich).

### Cytokine bead array assay

The concentrations of TNFα in sera and tumors were measured using mouse enhanced sensitivity cytokine bead array kit (BD Biosciences; USA) according to the manufacturer’s protocol. Serum was isolated by incubating blood samples at room temperature for 30 min and then centrifuging at 1500 g for 15 min at room temperature to collect the supernatant. To measure TNFα levels in tumors, tumor lysate was prepared as described above and was used at a 1:25 dilution in parallel with standards spiked with an equivalent amount of RIPA lysis buffer. The beads samples were analyzed on a FACS Canto (BD Biosciences), and the TNFα concentrations were calculated using FCAP array software (BD Biosciences).

### Tumor phenotyping and intracellular staining

Cells were isolated from tumors as described previously [26] and resuspended in media. A portion of cells was treated with 10 μg/ml of brefeldin-A (BFA) for 16 h in media alone or in media containing either 100 nM of GDC-0152 or 100 μg/ml of LPS. The remaining portion of untreated cells was used for cellular phenotyping. Cells were mixed with sorting buffer (PBS, 4% FBS, 5 mM EDTA) containing a cocktail of surface staining antibodies: CD49b(DX5)-PE, CD3-APC, Siglec-F-APC, F4/80-PE-Cy7, CD11c-V450, Ly6c-APC-Cy7, CD103-BV510 and Ly6G-BV711 (BD Biosciences) for 30 min at 4 °C, washed once with PBS and analyzed on a FACS ARIA III (BD Biosciences). mCherry fluorescence was used to identify tumor cells. For intracellular staining, samples treated with BFA were stained using the same antibody cocktail and then fixed with 1% paraformaldehyde for 15 min at room temperature in the dark. Samples were washed once with PBS and incubated with a TNFα-FITC antibody (BD Biosciences) in 0.4% saponin/PBS for 1 h at RT, washed and analyzed on a FACS ARIA III to detect TNFα positive cells co-stained with phenotyping markers. Flow cytometric data were analyzed using FCS Express (De novo Software; USA).

### Quantitative PCR

DNA was extracted from luciferase clones using the DNeasy Blood and Tissue Kit (Qiagen, Hilden, Germany) as per the manufacturer’s instructions. Left and right mouse lungs were separated and ground with a scalpel blade before being transferred to a tube containing digestion buffer (10 mM Tris-HCl, 1 mM EDTA, 1 mg/mL Proteinase K, 0.5% SDS). Samples were incubated for 24 to 36 h at 56 °C with shaking at 800 rpm until all tissue appeared visually to be dissolved. Digested lungs were vortexed for 10 s then washed twice in an equal volume of Phenol: Chloroform: Isoamyl Alcohol (25:24:1) and centrifuged at 13,000 g for 5 min at 4 °C. DNA was precipitated in an equal volume of isopropanol and 0.3 M sodium acetate and centrifuged at 13,000 g for 15 min at 4 °C. The DNA pellet was washed with 70% cold ethanol. DNA was resuspended in TE buffer (10 mM Tris-Cl, pH 8.0, 1 mM EDTA). DNA was quantitated using a NanoDrop 1000 and diluted prior to qPCR analysis with Milli-Q water. All qPCR assays were performed on a Bio-Rad C1000 thermocycler using Power SYBR green PCR master Mix (Thermo Fisher Scientific) in tear-away 96 well PCR plates. Primers designed to amplify luciferase DNA were GCAACCAGATCATCCCCGAC and GCTGCGCAAGAATAGCTCCT. Primers used to amplify part of the murine vimentin gene were AGCTGCTAACTACCAGGACACTATTG and CGAAGGTGACGAGCCATCTC [63]. All reactions contained 500 nM of each primer and 100 ng of template DNA, and used these conditions: 50 °C for 2 min, 95 °C for 2 min, then forty cycles of 95 °C for 15 s, 56 °C for 15 s, 72 °C for 1 min. Cycle threshold (Ct) values were set to 10 standard deviations from the mean fluorescence during cycles 5 to 15. Relative tumor burden (RTB) was calculated using the equation RTB = 10,000/2^ΔCt^, where ΔCt was the difference between the Ct values for luciferase and vimentin reactions [63]. GraphPad Prism software was used to calculate the amount of DNA present in unknown samples from standard curves that were generated using DNA extracted from KRIB-Luc cells serially-diluted into DNA isolated from lungs of tumor-free mice.

### Statistics

GraphPad Prism 8.0 was used to perform the statistical tests specified in the figure legends.

## Results

**Fig. 1:**
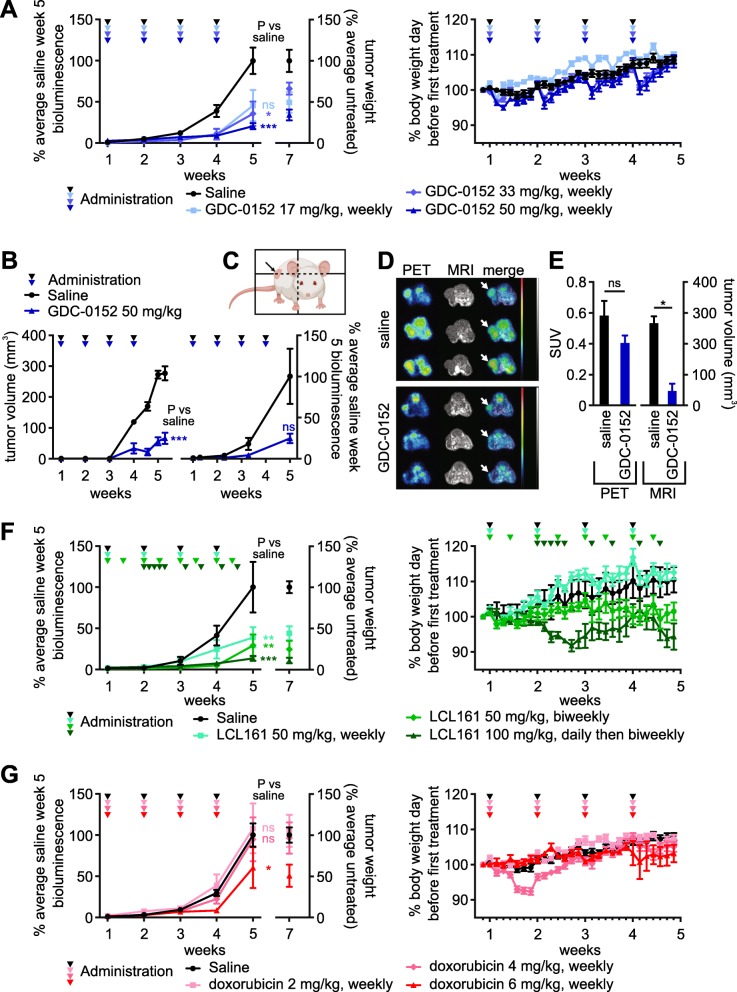
GDC-0152, LCL161 and doxorubicin impede the growth of subcutaneously implanted osteosarcomas in mice. Luciferase-expressing murine 1029H osteosarcoma cells were implanted subcutaneously into nude mice. 1 week after implantation the mice commenced the specified regimens of GDC-0152 (**a**-**e**), LCL161 (**f**) or doxorubicin (**g**). **a**, **f**, **g** Left panels: Primary tumor growth was monitored via bioluminescence, and tumor weights were measured post-mortem. One way ANOVAs with Sidak post-tests were used to estimate the probability that the drug treatments significantly affected tumor growth, as measured by bioluminescence at week 5, relative to saline treatment (*** *P* < 0.001; ** *P* < 0.01; * *P* < 0.05; ns *P* > 0.05 (colors of asterisks and “ns” labels; reflect the treatments, as indicated in the in-figure legends). Right panels: Mice were weighed each day to assess drug toxicity (*n* = 5–25, +/− SEM). **b**-**e** Tumor-bearing mice were treated with saline or GDC-0152 50 mg/kg/week. **b** Tumor responses were monitored by caliper measurements (left) or bioluminescence (right) at the indicated times (*n* = 3, +/− SEM). The differences between responses in saline and drug-treated mice were analyzed by a one way ANOVA with Sidak post-tests (*** *P* < 0.001). (C, D) ^18^F-FDG PET/MRI imaging was performed 30 days after the first treatment. **c** A cartoon, created using BioRender, illustrates the plane of transverse PET/MR images taken through 1029H osteosarcoma tumors (denoted by the arrow). **d** PET/MR imaging was performed on each mouse per treatment group (*n* = 3), oriented with the spine at the top and the femurs in the lower left and right parts of the images: *left*, positron emission tomography (PET); *middle*, magnetic resonance imaging (MRI); *right* column, PET/MRI overlay with white arrows indicating tumors . The color scale, which ranged from 0 to 1.5 SUV, indicates highest uptake of ^18^F-FDG in red and lowest uptake in black. The gray scale used for MR imaging, which ranged from 40.95 to 4095, indicates brightest signals from fat-containing soft tissues versus darker signals from water-containing soft tissues. (**e**) Mean standardized uptake values (SUV) of ^18^F-FDG-PET were determined by volume of interest (VOI) analysis, and tumor volumes were determined by VOI analysis of MRI images (*n* = 3, +/− SEM). Mann-Whitney non-parametric U-tests were used to calculate the significance of differences between treated and untreated mice * *P* < 0.05; ns *P* > 0.05

We previously profiled the in vitro sensitivity of cells from a number of spontaneous primary and metastatic murine osteosarcomas to a panel of Smac mimetics. SM-164, GDC-0152 and LCL161 potently sensitized cells from most tumors to killing by TNFα, although we observed some inter-tumor variability in the magnitude of this effect [26]. We generated luciferase- and mCherry-expressing derivatives of a subset of those murine osteosarcoma cell lines, to monitor tumor growth and drug responses in vivo. A reporter gene-expressing derivative of the murine osteosarcoma cell line 1029H, which displayed intermediate in vitro sensitivity [26], was reproducibly tumorigenic upon subcutaneous implantation into nude mice, so was selected for initial evaluation of the in vivo efficacy of Smac mimetics. Of the three Smac mimetics that cooperated most potently with TNFα to kill osteosarcoma cells in vitro, LCL161 and GDC-0152 have progressed furthest towards clinical use [19, 20, 66], so they were selected for pre-clinical in vivo anti-osteosarcoma testing. Bioluminescence readings during the first 5 weeks after implantation demonstrated that GDC-0152 strongly suppressed tumor growth (Fig. 1a). Bioluminescence readings were unreliable after this time, presumably reflecting poor uptake of luciferin into large tumors. Tumors were resected and weighed post-mortem to assess and compare the ultimate outcome of the treatments. Tumors regrew after GDC-0152 treatment ceased, as reflected by the weights of the tumors and the bioluminescence reading taken a week after the last drug administration. Caliper measurements, ^18^FDG-PET and MRI were also used to evaluate tumor responses to GDC-0152 treatment (Fig. 1b-e). Confirming the anti-osteosarcoma activity of GDC-0152 detected using bioluminescence and via tumor weights at endpoint (Fig. 1a), tumors in GDC-0152-treated mice were less metabolically active and significantly smaller than the untreated tumors (Fig. 1b-e). Mice given the highest dose of GDC-0152, 50 mg/kg, lost around 5% of their body weight the day after each drug delivery, but gradually recovered to attain similar weights to their untreated peers within a week of each treatment (Fig. 1a, right panel). This was a more pronounced adverse effect than that reported by Flygare et al., who only noted a reduction in body weight when tumor-bearing nude mice were given 100 mg/kg of GDC-0152 [19]. The likelihood that further dose escalation would have been intolerably toxic precluded us from testing whether a higher dose of GDC-0152 may have produced a more durable anti-tumor response.

LCL161 treatment also significantly impeded osteosarcoma growth (Fig. 1f). A published regimen (100 mg/kg each weekday) was very effective but, in contrast to a previous report that failed to detect any toxicity associated with this treatment [67], we observed substantial weight loss. After noting cumulative weight loss after the initial five daily administrations, we reduced the administration frequency to twice weekly, which prevented further net weight loss, but the animals failed to reach normal weights (Fig. 1f, right panel). The intermediate dosing regimen, 50 mg/kg twice per week, was slightly less effective but better tolerated, although this dosing prevented normal weight gain by these young animals.

Doxorubicin had less impact on osteosarcoma growth than the Smac mimetics in this model. Only the highest dose of 6 mg/kg/week significantly impaired tumor growth (Fig. 1g). This was counter-intuitive, given the clinical efficacy of doxorubicin for treating osteosarcoma patients [68], and the in vitro sensitivity of 1029H cells to this agent [26]. Doxorubicin has been documented to penetrate poorly into tumors [69] so it is possible the marginal efficacy of doxorubicin in this context reflects a low bioavailability of this poorly penetrant drug within subcutaneous tumors that may not be extensively vascularized [70].

**Fig. 2:**
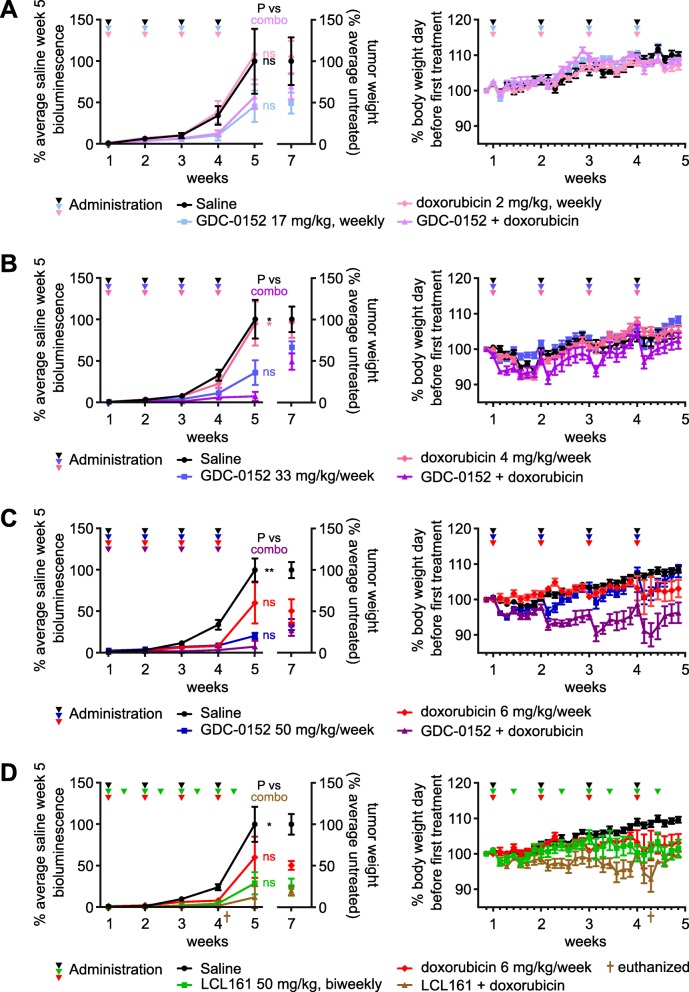
Co-treatment with GDC-0152 or LCL161 plus doxorubicin suppresses the growth of subcutaneously implanted osteosarcomas in mice. Luciferase-expressing murine 1029H osteosarcoma cells were implanted subcutaneously into nude mice. 1 week after implantation the mice commenced the specified regimens of GDC-0152 (**a**-**c**) or LCL161 (**d**) and/or doxorubicin. Left panels: Primary tumor growth was monitored via bioluminescence, and tumor weights were measured post-mortem. One way ANOVAs with Sidak post-tests were used to compare tumor growth 5 weeks after commencing combination versus sole agent or saline treatments (** *P* < 0.01; * *P* < 0.05; ns *P* > 0.05;colors of asterisks and “ns” labels reflect the treatments, as indicated in the in-figure legends). Right panels: Mice were weighed each day to check for drug toxicity (*n* = 5–25, +/− SEM). (**d**) One mouse that was administered LCL161 plus doxorubicin lost more than 15% of its starting weight so was euthanized

On average, tumor growth was more substantially hampered by co-treatment with medium to high doses of Smac mimetics and doxorubicin than by the drugs as sole agents (Fig. 2a-d, left panels), although tumors regrew after treatment cessation. Although this trend of cooperation was observed in multiple experiments, statistical analyses failed to rule out the possibility that these differences were due to chance. The suggestion of an improvement in efficacy associated with the co-treatment was however accompanied by enhanced toxicity (right panels). One mouse that received twice-weekly treatment with 50 mg/kg LCL161 plus weekly administration of 6 mg/kg doxorubicin lost more than 15% of its weight within a day, necessitating euthanasia. In subsequent experiments involving co-treatment with these drugs, we therefore reduced the frequency of LCL161 administration from twice-weekly to weekly.

**Fig. 3:**
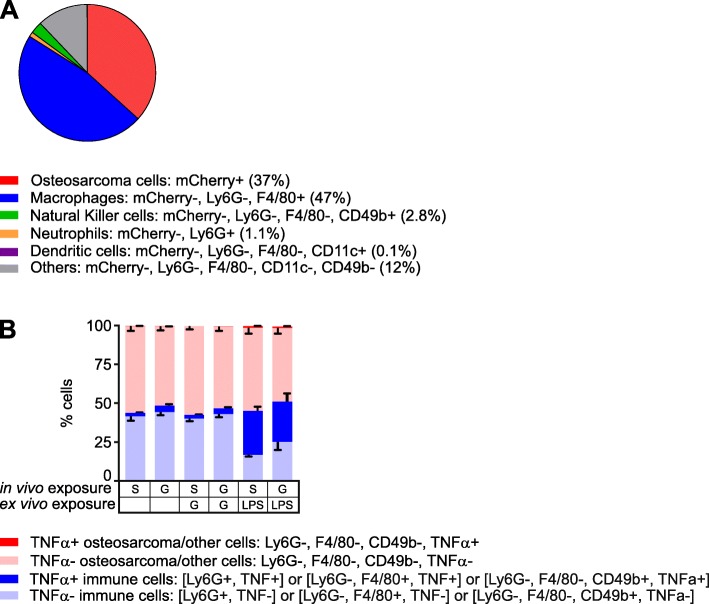
Tumor-infiltrating immune cells produce TNFα within implanted osteosarcomas in mice. **a** Disaggregated unfixed 1029H-Luc subcutaneous tumor cells were phenotyped by flow cytometry. mCherry-expressing cells were designated osteosarcoma cells; these lacked markers for myeloid and NK cells. Immunophenotyping identified macrophages, dendritic cells, neutrophils and natural killer cells. No cells expressed detectable Siglec-F, CD103, CD3 or Ly6C markers. **b** Tumors from mice treated with saline (S) or GDC-0152 50 mg/kg (G) were harvested and disaggregated. Cells were incubated in media containing brefeldin-A, with or without 100 nM GDC-0152 (G) or 100 μg/ml LPS (LP), then incubated with a panel of antibodies recognizing cell type markers (as in panel **a**), fixed and then stained for TNFα. mCherry fluorescence was not detected after fixation, so unstained cells were designated as “osteosarcoma or other”. Positively identified neutrophils, macrophages and natural killer cells are grouped as “immune cells”. The percentage of cells of each type in each sample, expressing and lacking TNFα were calculated (*n* = 3, +/− SEM)

Although GDC-0152 and LCL161 could theoretically kill cells via relieving XIAP-mediated caspase inhibition, their major mechanism of lethality involves stimulation of cIAP1/2 degradation, facilitating RIPK1 de-ubiquitination, which redirects TNFα-mediated TNFR1 signaling towards apoptotic or necroptotic pathways [71]. Unlike some other cell types that can produce autocrine TNFα in response to Smac mimetic treatment [23–25], this class of drugs only killed osteosarcoma cells upon provision of exogenous TNFα [26]. The in vivo efficacy of GDC-0152 and LCL161 we observed in this study therefore implied either that the in vivo tumor microenvironment somehow imbued osteosarcoma cells with the ability to produce autocrine TNFα, or that host cells in or around the tumors secreted TNFα that cooperated with the administered Smac mimetics to kill the osteosarcoma cells in vivo. Our data support the latter model. Flow cytometry revealed that only 37% of the cells comprising a subcutaneous tumor expressed detectable mCherry fluorescence. Around half of the cells within this tumor were infiltrating host cells, mostly macrophages (Fig. 3a). The phenotypes of 12 % of the cells could not be determined with the antibody panel we used; some were probably osteosarcoma cells whose mCherry fluorescence was too weak to detect and others were probably other types of infiltrating host cells. We performed intracellular cytokine staining of fixed tumor cells from three untreated mice and three animals that received a single dose of GDC-0152 six hours prior to culling. Unfortunately, the fixation abolished mCherry fluorescence, so 1029H-Luc cells could not be distinguished from other cells that lacked markers detected by our antibodies. Approximately 2–4% of the cells within tumors, mostly immune cells, produced TNFα, and this proportion was very slightly higher in samples from mice that received GDC-0152 treatment (Fig. 3b). Hardly any cells that lacked immune cell markers, which presumably were mostly 1029H-Luc osteosarcoma cells (Fig. 3a), contained TNFα (Fig. 3b). Ex vivo incubation of the tumor cells with lipopolysaccharide (LPS), but not GDC-0152, induced the majority of the immune cells to express TNFα (Fig. 3b).

**Fig. 4:**
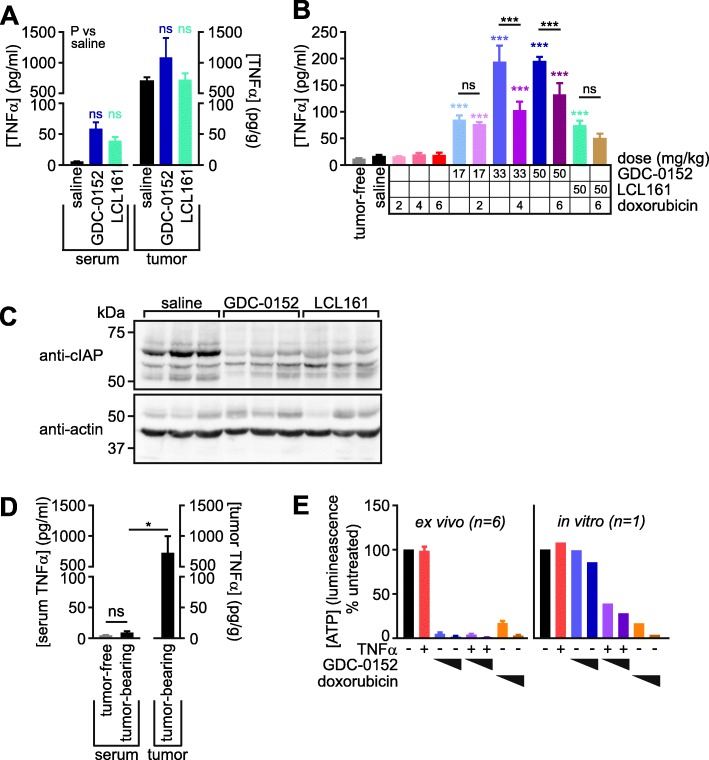
Implanted and spontaneous osteosarcomas contain high concentrations of TNFα. **a** Seven weeks after subcutaneous 1029H-Luc implantation, mice were administered a single dose of saline, GDC-0152 (50 mg/kg) or LCL161 (50 mg/kg). Six hours later, the mice were culled and their blood and tumors were harvested. Serum and tumor lysates were prepared and TNFα levels were measured and used to calculate TNFα abundance per milliliter of serum or per gram of tumor. One way ANOVAs and Sidak’s post-tests were used to determine if the treatments significantly influenced TNFα levels in blood or tumors (*P* > 0.05 for all comparisons; *n* = 5, +/− SEM). **b** TNFα was quantitated in the serum of mice 6 h following the final administration (after 4 weeks of treatment) of the listed agents to tumor-bearing mice, or tumor-free untreated mice. One-way ANOVA analyses with Sidak’s post-tests were used to estimate the probability that random chance accounted for the differences observed between saline-treated mice and those treated with drugs or tumor-free animals (colored asterisks), and whether doxorubicin significantly altered the TNFα responses to Smac mimetics (black asterisks and “ns” labels) (*** *P* < 0.001; ns *P* > 0.05; *n* = 3–11, +/− SEM). **c** Lysates from tumors resected from treated and untreated mice were immunoblotted using an antibody that detects both cIAP1 (70 kDa) and cIAP2 (67 kDa). Loading was visualized by immunoblotting for beta actin (42 kDa). **d** TNFα was quantitated in the serum and tumors of four tumor-bearing *Osx*-Cre p53^*fl/fl*^ pRb^*fl/fl*^ mice, and in the serum of three tumor-free p53^*fl/fl*^ pRb^*fl/fl*^ mice. A one-way ANOVA analysis with Sidak’s post-tests was used to estimate the probability that random chance accounted for the differences in TNFα concentrations between the blood of the tumor-bearing mice versus either their tumors or the blood of tumor-free animals (* *P* < 0.05; ns *P* > 0.05; *n* = 3–4, +/− SEM). **e** 1029H-Luc tumors were resected from six untreated mice. The cells were disaggregated, then cultured alongside in vitro-cultured 1029H-luc cells for 48 h in media containing no drugs, 1 μM or 3 μM doxorubicin, 100 pg/ml murine TNFα and/or 1 μM or 10 μM of GDC-0152. Residual ATP was quantitated using CellTitreGlo (*n* = 6 +/− SEM for resected tumors)

High concentrations of TNFα, presumably derived from tumor-associated immune cells, were detected within lysates of tumors resected from mice six hours after administration of a single dose of saline, GDC-0152 or LCL161 (Fig. 4a). If most of the TNFα in these tumors was within interstitial fluid, and this constituted around 10% of the tumor volume (as was reported for subcutaneous fibrosarcomas [72]) our data suggest that the tumor cells in this implantation model may be exposed to around 6–10 pg/ml of TNFα in vivo, a concentration that achieved approximately half-maximal cooperation with Smac mimetics to kill osteosarcoma cells in vitro [26]. Analysis of blood harvested six hours after either a single drug treatment (Fig. 4a) or the last of four weekly treatments (Fig. 4b) confirmed published observations [20, 73] that these drugs dramatically increased levels of TNFα in the blood. This effect, which was particularly pronounced for GDC-0152, was ameliorated by co-treatment with doxorubicin (Fig. 4b), reflecting its established myelosuppressive activity in humans [74] and mice [75]. In vivo treatment with GDC-0152 or LCL161 reduced tumor levels of cIAP1/2, confirming the drugs accessed the tumors and exerted their expected biochemical effect on tumor cells (Fig. 4c). The presence of macrophages in spontaneously-arising osteosarcomas has been published [58–60], but to our knowledge the amount of TNFα within naturally-arising osteosarcomas have not previously been measured. To investigate TNFα levels in spontaneous osteosarcomas within immunocompetent animals, we harvested tumors and blood from mice that developed osteosarcomas due to an osteoblast lineage-specific deletion of the tumor suppressor genes p53 and Rb [65]. Blood from tumor-free animals was also collected for comparison. The spontaneous osteosarcomas, like the subcutaneously implanted tumors, contained abundant TNFα (Fig. 4d). In keeping with our observation that the anti-osteosarcoma potential of Smac mimetics hinges on the presence of TNFα produced by myeloid cells within the tumors, disaggregated cells from freshly-resected implanted tumors (consisting of both osteosarcoma and infiltrating non-cancerous cells) were efficiently killed in vitro by Smac mimetics as sole agents, whereas the corresponding in vitro-cultured osteosarcoma cells were only sensitive to Smac mimetics when co-treated with exogenous TNFα (Fig. 4e).

**Fig. 5:**
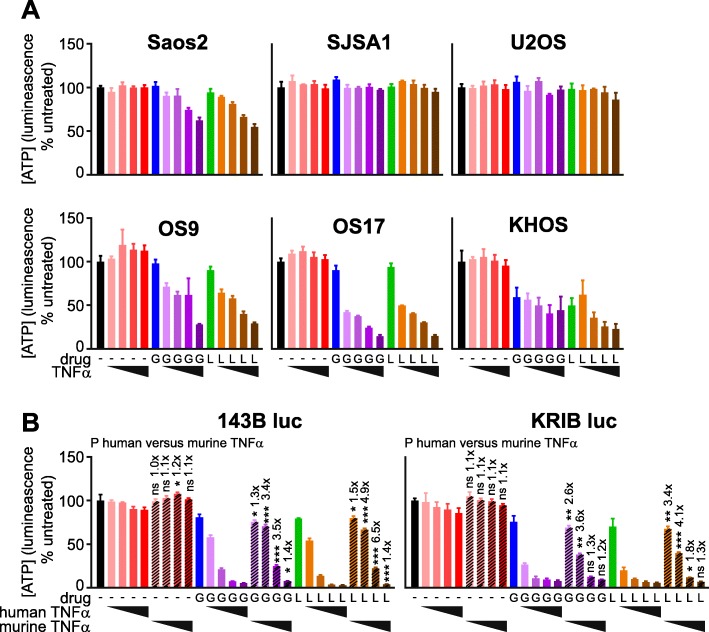
Human osteosarcoma cell lines vary in sensitivity to Smac mimetics +/− TNFα in vitro. Parental (**a**) or luciferase-expressing (**b**) human osteosarcoma cell lines were incubated for 48 h in media containing 0, 1, 10, 100 or 1000 pg/ml human (**a**, **b**) or murine (**b**) TNFα and/or 3 μM GDC-0152 (“G”) or 3 μM LCL161 (“L”). Residual ATP was quantitated using CellTiter-Glo (*n* = 3 +/− SEM). (**b**) T tests with Holm-Sidak corrections for multiple comparisons were used to determine the likelihood that random chance accounted for the differences observed between responses to human versus murine TNFα, for each cell line alone or in conjunction with GDC-0152 or LCL161. (*** *P* < 0.001; ** *P* < 0.01; * *P* < 0.05; ns *P* > 0.05; *n* = 3, +/− SEM). The numbers above the *P* value data indicate the ratio of luminescence (as a surrogate for survival) of cells treated with each concentration of murine versus human TNFα, alone and together with the Smac mimetics

We were interested in whether cells from human osteosarcomas would exhibit similar Smac mimetic sensitivity profiles to their murine counterparts, in vitro and in vivo. To explore this, we determined the in vitro sensitivity of a panel of human osteosarcoma cell lines to GDC-0152 or LCL161, alone or with TNFα, using the “CellTiter-Glo” assay. In this assay, a reagent containing high concentrations of luciferase plus its substrate luciferin is applied to treated or untreated cells. The intensity of light emitted correlates with the amount of ATP in the well, which enables luciferase to catalyse the luminescent reaction. Two minimally passed human osteosarcoma cell lines, OS9 and OS17 [62], survived incubation with Smac mimetics as sole agents but responded to co-treatments with TNFα (Fig. 5a), like cells from most of the murine tumors we previously tested [26]. The responses of established human osteosarcoma cell lines (SaOS2, SJSA1, U2OS, 143B and KRIB) varied substantially, however. SJSA1 and U2OS were resistant, even to co-treatment with Smac mimetics plus TNFα. KHOS cells were somewhat sensitive to Smac mimetics alone, and addition of TNFα only slightly augmented this sensitivity. SaOS2 cells were slightly less sensitive than OS9 and OS17 to co-treatment with Smac mimetics and TNFα. Parental and luciferase-expressing derivatives of 143B and KRIB were slightly more sensitive to the combination treatment than OS9 and OS17 (Fig. 5b, data not shown). To model the expected exposure of the human tumor cells to Smac mimetics and TNFα following implantation into nude mice, we compared the extents to which Smac mimetics sensitized the luciferase-tagged KRIB and 143B human osteosarcoma cells to murine versus human TNFα. The CellTiter-Glo assay was used for these experiments. The CellTiter-Glo reagent was designed to contain sufficient luciferase to ensure that reaction rates are proportional to ATP concentrations across a large range of cell densities, so we suspect that the additional presence of some transgene-encoded luciferase in these cells would be unlikely to affect the reaction rate and hence the light emitted. However we cannot conclusively exclude the possibility that lower luminescent readings following drug treatment may reflect a reduction in cellular luciferase, as well as ATP levels, as cells died. Although published data suggest human TNF receptors bind murine TNFα with only slightly lower affinity than human TNFα [76–78], the human osteosarcoma cells were significantly more sensitive to Smac mimetics coupled with human than murine TNFα (Fig. 5b).

Our observation that doxorubicin only slightly impaired the growth of subcutaneously implanted murine osteosarcomas raised the possibility that vascularization of these tumors may be poor, despite obviously being sufficient to mediate intratumoral access of immune cells and Smac mimetics. We therefore decided to use a different implantation route for testing in vivo drug efficacy against human osteosarcomas. We first considered orthotopic routes. Intrafemoral and intratibial osteosarcoma implantation models have been developed, but the technical challenges associated with these procedures can lead to highly variable tumorigenicity rates, and intraosseous tumors are not well tolerated by mice [79–81]. These factors would have necessitated using large numbers of animals to discern significant drug effects, and the need to provide analgesia could have introduced potentially confounding drug-drug interactions. Given the requirement of the inflammatory cytokine TNFα for the anti-osteosarcoma activity of Smac mimetics, we were particularly keen to avoid analgesics with anti-inflammatory activities. We therefore decided to establish an intramuscular implantation model for testing the impact of Smac mimetics on human osteosarcoma xenografts. Intramuscular implantations of osteosarcoma cells, either into the upper hind paw [82] or gastrocnemius muscle [83, 84], were reported to be highly tumorigenic. To minimise the tumors’ impact on leg function, we chose to inject luciferase-expressing KRIB human tumor cells into the cranial tibial muscle of mice. This yielded reproducible primary tumor growth that was well tolerated by the mice (obviating the need for analgesia), and metastases to the lungs of all untreated mice within 7 weeks of implantation.

**Fig. 6:**
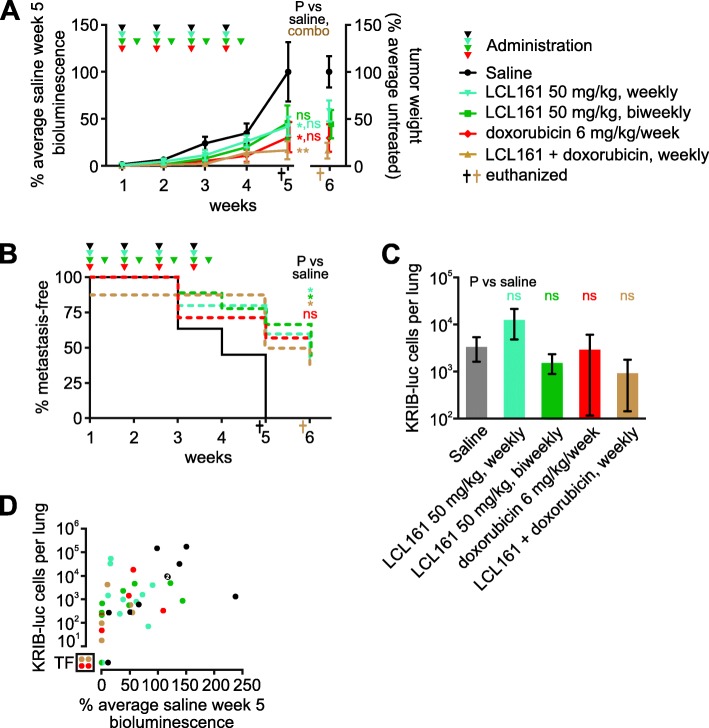
LCL161 reduces primary and metastatic growth of human osteosarcoma cells in mice. Luciferase-expressing human KRIB osteosarcoma cells were implanted intramuscularly into nude mice. One to 2 weeks after implantation the mice were administered the specified treatments. **a** Primary tumor growth was monitored via bioluminescence. Tumor material that could be confidently resected from the surrounding muscle post-mortem was weighed. Some data points at the 5 and 6 week timepoints were slightly horizontally offset to enable all to be visible. A one way ANOVA with Sidak’s post-tests was used to estimate the probability that the drugs significantly affected tumor growth 5 weeks after treatment commenced, and whether the response to co-treatment differed significantly from responses to weekly administration of LCL161 or doxorubicin as sole agents (** *P* < 0.01; * *P* < 0.05; ns *P* > 0.05; *n* = 7–11, +/− SEM). **b** The times at which luminescence was first detected in the lungs were recorded. Log-rank (Mantel-Cox) tests were used to compare the onset of metastases in untreated mice versus animals that received each treatment, excluding one co-treated mouse that already had detectable metastatic disease prior to the first treatment. Bonferroni correction was used to adjust the resulting *P* values for multiple (4) comparisons (* *P* < 0.05; ns *P* > 0.05; *n* = 7–11, +/− SEM). **c** Lung tumor burden at endpoint was determined by quantitative PCR for surviving mice (excluding one untreated and one-co-treated that had already been euthanized). The assay reliably detected > 10 KRIB-Luc cells per lung. A one way ANOVA with Sidak’s post-tests was used to estimate the probability that the drugs significantly affected lung tumor burden (ns *P* > 0.05; *n* = 7–10, +/− SEM). **d** Metastatic burden was compared with bioluminescence at week 5 (the most reliable measure of primary tumor growth), for each mouse. Data from each mouse is represented by a circle colored to reflect its treatment. Some circles have been cropped to ensure they are all visible. Two saline-treated mice had very similar metastatic and primary tumor burdens; denoted by a white “2” superimposed on those overlapping circles. One saline-treated mouse (denoted by the bottom black dot in the graph) developed detectable lung bioluminescence during the experiment but lacked detectable luciferase DNA within its lungs at the endpoint. Circles in the bottom left box (labeled “TF”) signify tumor free mice. These animals lacked detectable bioluminescence 5 weeks after treatment commenced, no primary tumors were visible upon dissection 1 week later, and they also lacked detectable luciferase DNA in their lungs

As mentioned above, KRIB cells were only sensitive to Smac mimetics in vitro in the presence of exogenous TNFα, and murine TNFα cooperated with these drugs less potently than human TNFα in vitro, implying that this xenograft model may underestimate the ability of Smac mimetics to eliminate human osteosarcoma cells in patients. Nevertheless, LCL161 limited the growth of intramuscular KRIB tumors (Fig. 6a). Doxorubicin was also effective in this model, and co-treatment was very effective (Fig. 6a). This model enabled monitoring of metastases development, as measured by in vivo lung bioluminescence (Fig. 6b) and qPCR-based quantitation of the lung tumor burden at the experimental endpoint (Fig. 6c). Weekly or twice-weekly LCL161 administration, and weekly co-treatment with LCL161 plus doxorubicin significantly delayed metastases development (Fig. 6b). The numbers of osteosarcoma cells within the lungs of mice within each treatment group varied substantially (Fig. 6c, d) so, although LCL161 administration or co-treatment with doxorubicin slowed metastasis development (Fig. 6b), we did not discern statistically significant effects of treatment on ultimate lung tumor burden (Fig. 6c). Two mice treated with doxorubicin and two co-treated with LCL161 plus doxorubicin failed to develop pulmonary metastases and experienced durable primary tumor regressions: primary tumors were undetectable from week 3 for the two co-treated mice and from weeks 4 and 5 for those two doxorubicin-treated animals (Fig. 6d).

## Discussion

**Fig. 7:**
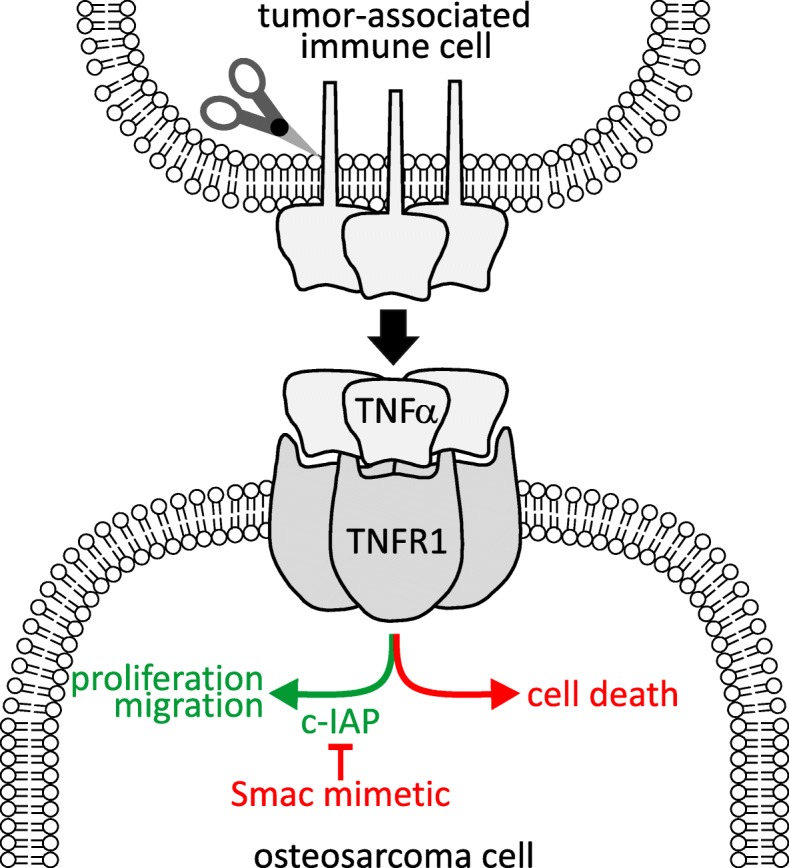
Model for Smac mimetic anti-osteosarcoma efficacy. Smac mimetic treatment induces osteosarcoma cells to activate TNFR1-mediated cell death pathways in response to TNFα produced by intratumoral immune cells

These experiments revealed that Smac mimetics GDC-0152 and LCL161 impeded the growth of implanted osteosarcomas in nude mice. The in vitro sensitivity of the murine and human osteosarcoma cells used to create these tumors depended on supplied TNFα. The in vivo efficacy we observed was probably due to high levels of endogenous TNFα within the implanted tumors (Fig. 7). This introduced a slight complication into our experiments designed to test Smac mimetic efficacy against human osteosarcoma cells grown in mice: murine TNFα cooperated with Smac mimetics less potently than human TNFα, thus our mouse experiments may have underestimated the potential for LCL161 to treat osteosarcomas in patients. Like the implanted tumors, spontaneously arising osteosarcomas that arose in genetically engineered immunocompetent mice also bore high concentrations of TNFα, excluding the possibility that this phenomenon was an artefactual consequence of tumors implanted into nude mice. Immunophenotyping revealed that implanted osteosarcomas, like patient tumors [58–60], were heavily infiltrated by immune cells, which our data suggest were responsible for producing most of the intratumoral TNFα. Although we did not formally test the requirement for TNFα in order for Smac mimetics to exert anti-osteosarcoma effects in our model, this conclusion is consistent with our data showing that (a) Smac mimetic sensitivity of osteosarcoma cells in vitro depended on exogenous TNFα, (b) Smac mimetics retarded growth of tumors derived from these cells in vivo, and (c) implanted osteosarcomas contained TNFα that was produced by intratumoral immune cells. We would predict that Smac mimetic treatments would be ineffective in osteosarcoma-bearing animals treated with TNFα-blocking agents, or TNFα-deficient mice. Indeed, the presumed deficiency in TNFα-producing tumor-associated myeloid cells within SCID mice probably explains the relatively poor anti-osteosarcoma efficacy of LCL161 in treating SCID mice bearing patient-derived xenografts [21]. Mice bearing implanted or spontaneous osteosarcomas had around twice as much TNFα in their blood as tumor-free animals. Although this difference was not statistically significant, it mirrored published data from humans: the TNFα concentration in sera of osteosarcoma patients was approximately double that in control individuals’ blood [56]. This implies that osteosarcoma cells within patients’ tumors may be exposed to enough TNFα to render them sensitive to the lethal effects of Smac mimetics, but direct measurement of TNFα within patients’ tumors would be necessary to confirm this suspicion.

Doxorubicin, the linchpin of current osteosarcoma regimens, exhibited marginal single agent efficacy against subcutaneously-implanted 1029H tumors, but was more effective at reducing the growth of intramuscularly-implanted KRIB tumors. In both contexts, doxorubicin tended to cooperate with Smac mimetics to retard tumor growth, although this trend was not statistically significant. This co-treatment was particularly effective and sustained in the KRIB intramuscular model. Encouragingly, LCL161, alone or with doxorubicin, also significantly delayed the appearance of pulmonary metastases in mice bearing KRIB intramuscular tumors. Further work will be needed to ascertain whether this anti-metastatic effect was because the treated mice had smaller primary tumors (which would presumably seed fewer tumor cells to the lungs) and/or reflected drug-mediated destruction of osteosarcoma cells located within the lungs.

Subsequent studies will also be required to accurately model the potential benefit of co-treatment of osteosarcoma patients with Smac mimetics plus doxorubicin, versus single agent treatment, and to consider the balance between efficacy versus toxicities conferred by co-treatment, relative to Smac mimetics alone or coupled with other chemotherapy drugs. Doxorubicin dampened the inflammatory effect of Smac mimetic treatment, as reflected in less drastically elevated serum TNFα levels in co-treated mice, relative to animals that just received GDC-0152, consistent with the established myelosuppressive activity of doxorubicin [74, 75]. Despite that amelioration of the Smac mimetics’ dose-limiting toxicity, mice that received doxorubicin plus Smac mimetics lost more weight than animals that only received one drug. Additional research will be needed to determine the mechanism underlying this cooperative toxicity, including exploration of the possibility that Smac mimetics may exacerbate doxorubicin’s cardiotoxicity [85]. It will be important to determine whether cooperative toxicities would be avoided by sequential exposure, in which case subsequent Smac mimetic treatment may be considered for patients whose tumors persist or recur after administration of the maximal cumulative dose of doxorubicin recommended to avoid dose-limiting cardiotoxicity. The outcome of those experiments may help define clinical contexts in which the anti-osteosarcoma efficacy of Smac mimetics could be maximized while their toxicities are managed.

The clinical responsiveness of individual osteosarcomas to Smac mimetics will presumably be strongly dictated by the ability of the drugs to cooperate with TNFα to trigger apoptotic or necroptotic death of the individual’s cancer cells. Further work will be needed to gain a comprehensive understanding of intertumoral variability in the in vitro sensitivity of human osteosarcomas to Smac mimetics. Cells from two minimally-passaged human osteosarcomas were quite sensitive to Smac mimetic/TNFα co-treatment, however established human osteosarcoma cell lines varied substantially in their sensitivity to Smac mimetics as sole agents and together with TNFα. This heterogeneity may reflect biological variability between different tumors, and/or it may be a consequence of genomic instability-mediated phenotypic drift during extended in vitro culturing [86]. If the latter is a major factor, the sensitive phenotypes of the minimally passaged lines (OS9 and OS17) may reflect the typical responsiveness of osteosarcoma cells within patients’ tumors better than the established cell lines, some of which were more resistant.

It is important to note that our experiments were conducted in nude mice. Although these mice possess innate immune cells, which can produce the TNFα required for Smac mimetic-mediated osteosarcoma cell destruction, they have almost no T cells [87]. If Smac mimetics can stimulate immune-targeting of osteosarcoma cells through boosting lymphocyte survival and activation, as has been demonstrated in other cancers [48], this may augment the direct osteosarcoma cell killing we observed in nude mice, to yield a more pronounced anti-osteosarcoma effect in immunocompetent animals or humans.

## Conclusions

The Smac mimetics LCL161 and GDC-0152 cooperated with TNFα produced by infiltrating immune cells to limit osteosarcoma growth and metastasis in nude mice. These data illustrate the potential for Smac mimetics to target malignancies like osteosarcoma in which the cancer cells fail to produce autocrine TNFα in response to these agents. Results from this study suggest that safe regimens involving Smac mimetics like LCL161 or GDC-0152 may improve treatment outcomes for osteosarcoma patients.

## Data Availability

The datasets used and/or analyzed during the current study available from the corresponding author on reasonable request.

## Abbreviations

BCA: Bicinchoninic acid
BFA: Brefeldin-A
FBS: Fetal bovine serum
LPS: Lipopolysaccharide
MRI: Magnetic Resonance Imaging
PET: Positron Emission Tomography
